# Piloting the integration of SMART Recovery into outpatient alcohol and other drug treatment programs

**DOI:** 10.1186/s13722-023-00406-w

**Published:** 2023-09-06

**Authors:** V. Manning, A. D. Roxburgh, M. Savic

**Affiliations:** 1 Monash Addiction Research Centre, Eastern Health Clinical School, Monash University, Clayton, Australia; 2 Turning Point, Eastern Health, Box Hill, Australia

**Keywords:** Mutual aid, Addiction, Recovery, SMART Recovery, Peer support

## Abstract

**Background:**

Research suggests peer support groups can amplify and extend treatment effects and enhance long-term recovery from Alcohol and Other Drug (AOD) problems. However, they are rarely integrated into outpatient treatment programs, resulting in a missed opportunity for peer-to-peer learning, and increased connection to others social networks where people want to reduce or cease substance use.

**Method:**

In this mixed-methods study, we examined the uptake, participant experiences and impacts of Self-Management and Recovery Training (SMART) when embedded in three public AOD treatment programs in a pilot program in Australia. Groups were delivered initially in-person but transitioned online during the COVID-19 pandemic.

**Results:**

A total of 75 SMART Recovery groups were run by the pilot sites, with an average attendance of 6.5 people per meeting. Among Participants (*N* = 31) who completed the survey, 94% reported benefits relating to substance use (i.e., reduction/ successful maintenance of abstinence), 71% reported improvements in their mental health and wellbeing, 74% reported improvements in their physical health, and 81% reported feeling better connected with others. In-depth qualitative interviews were conducted with 10 participants to explore their experiences. Thematic analysis revealed four themes: motivation to attend, active ingredients, views on the integration of SMART into formal AOD, and the advantages and disadvantages of online groups.

**Conclusion:**

Taken together, these findings suggest embedding SMART Recovery in AOD treatment is a worthwhile endeavour. This was indicated by a good uptake and evidence of multiple and unique benefits to participants over and above their usual care, notably, better management of their AOD use, health, wellbeing, and sense of connection with others.

Peer support is the process of giving and receiving non-professional assistance from individuals with similar conditions or circumstances and has been shown to support the achievement of long-term recovery from psychiatric, alcohol, and/or other drug-related (AOD) problems [23, 32]. Peer support groups such as Alcoholics Anonymous (AA) can help maintain treatment gains, are widely available and are cost-effective [17]. The most well-known peer support groups, such as Alcoholics Anonymous (AA) and Narcotics Anonymous (NA), use the 12-step program, which promotes lifelong abstinence [11]. A recent Cochrane review [17] concluded that 12-step facilitation (professionally-delivered treatments that facilitate AA involvement) may be more effective than clinician administered treatments such as Cognitive Behaviour Therapy (CBT) in increasing rates of abstinence from alcohol. In addition, both manualised and non-manualised twelve step-facilitation was found to be equally effective when compared to clinician administered treatments in increasing other alcohol-related outcomes (e.g., drinking intensity) and alcohol-related consequences (e.g., physical, interpersonal, social consequences). The reviewers concluded that AA groups and 12-step facilitation are important and cost-effective treatments.

The positive impact of assertive linkage (i.e., actively introducing clients to meetings during formal treatment) to 12-step groups, in terms of subsequent attendance and outcomes, is long established [8, 21, 29, 31, 33], yet remains rarely implemented. Whilst people are able to attend peer-support groups in the community, in the Australian AOD treatment system, peer support groups only routinely feature as part of residential rehabilitation, which accounts for only 13% of all treatment episodes in 2020–2021 [2]. The value in attending peer support following initiation of a treatment episode was demonstrated in Australia’s largest treatment outcome study ‘Patient Pathways’ (n = 796) where attendance significantly increased the likelihood (OR = 1.72) of treatment success (defined as a clinically meaningful reduction in use or abstinence from their primary drug of concern), and with higher rates of treatment success among those attending more frequently [22]. As such, the authors recommend in a final report [20] that "*Specialist AOD services should develop and promote interventions and pathways to aftercare such as supportive community groups, including but not restricted to mutual aid groups*” (p. 16). Further, a recent study showed that frequent attendance at peer support groups was associated with greater treatment completion and retention [24]. With no restrictions on how often or how long someone can attend, strengthening pathways into peer-support groups from formal treatment services is particularly worthwhile given the lack of resources and high unmet need within current treatment systems [28].

The Australian AOD treatment system has long adopted a predominantly strengths-based and harm reduction approach to managing AOD problems [10, 25]. In contrast to the emphasis on accepting powerlessness over one’s addiction within the 12-step fellowship, SMART Recovery, adopts a strengths-based, person-centred approach drawing on evidence-based CBT and Motivational Interviewing techniques. Centred around a four-point program (building and maintaining motivation, coping with cravings and urges, problem solving, and lifestyle balance), SMART groups focus on developing and monitoring a plan for the week ahead with respect to an achievable goal to progressing recovery of an addictive behaviour. Furthermore, compared to the abstinence-orientation of 12-step groups, SMART Recovery aims to help people achieve their personal goals [14], which, in Australian meetings embraces abstinence as well as moderation, reductions, and other positive behaviour change [30]. In this way, SMART is highly compatible with most publicly-funded, professional treatment offered in Australia. In contrast to 12-step groups which are entirely peer led, SMART Recovery can be facilitated by peers with lived-experience, clinicians or non-clinicians who have completed SMART Recovery Training.

Research on SMART Recovery is still emerging. A systematic review of the effects of SMART Recovery for problematic alcohol use was unable to draw clear conclusions about its effectiveness due to the lack of quality studies, though the authors noted that the one RCT identified did support the benefits of SMART [4, 13]. In a RCT comparing an online alcohol intervention, with in-person SMART Recovery meetings and the two combined, all groups significantly reduced their percentage of days abstinent from alcohol and average drinks per day at the 3-month follow-up. However a dose–response was only detected for these outcomes (as well as reduced alcohol-related problems) in the SMART Recovery only group [13]. Zemore et al. [35] compared SMART Recovery to 12-step, and showed that, 12-step involvement was associated with better drinking related outcomes, but that this was entirely mediated by abstinence goals of those who attend 12-step, suggesting that the two have equivalent efficacy but attract different groups. The limited quantitative [1, 26, 35], qualitative [12], and mixed methods [18] research to date suggests SMART Recovery is highly valued by participants and leads to positive outcomes such as a reduction in the severity of dependence and improvement in psychological wellbeing [18], though rigorous RCTs are still lacking.

Due to the alignment in both philosophy and therapeutic approaches underpinning SMART Recovery and local government funded treatment services, we piloted the integration of SMART Recovery into three outpatient government-funded AOD services in the state of Victoria, Australia to determine client uptake, impact and experiences. The research questions were: (i) is there demand for SMART Recovery groups among clients attending AOD services (indicated by number of people attending each group), (ii) in what ways (if any), do clients benefit from attending (indicated through quantitative and qualitative responses), and (iii) what were clients’ experiences of attending service-run SMART Recovery groups, in addition to usual treatment, both online and in-person.

## Materials and methods

### Study design

In order to address the research aims/questions, we undertook a mixed-methods exploratory study involving a survey and qualitative interviews with people who attended SMART groups at three AOD treatment services.

### Setting and procedure

The pilot was run in Victoria, Australia in 2020 across three AOD treatment services (spanning both rural and metro areas and including one forensic service) that were selected from 20 services that submitted an expression of interest in participation. The three services were selected following an email invitation circulated via the states peak AOD body (Victorian Alcohol and Drug Association; VAADA), and based on selection criteria (i.e., demonstrated capacity and commitment). Ten facilitators (8 clinicians and 2 peers; 7 females and 3 males), who worked for the pilot services were identified by their service and selected to undertake facilitator training through SMART Recovery Australia before running groups. Groups had at least 2 facilitators (one peer and one clinician where possible) and ran for 90 min. Facilitators guided meetings to ensure consistent structure, adherence to group rules/guidelines and to encourage active participation of all members. Topics of discussion and peer-support emerged through the interactions between participants. Groups were delivered at the discretion of the service provider using the service’s own resources (including staff, facilities, and management support) and were offered to clients. Those attending the SMART Recovery groups were invited to complete a participant survey at the end of each meeting. Completion of surveys was voluntary and survey responses were anonymous. Surveys were initially distributed by facilitators in face-to-face groups directly to participants. After moving to online groups (due to COVID19), a new briefer online version of the survey was created, and the link distributed to participants via the Zoom chat function. Facilitators invited those who completed the survey to participate in a one-on-one qualitative interview with a researcher. Qualitative interviews were undertaken, audio-recorded, and then transcribed. Transcripts were then double checked by a different researcher for accuracy. Interviews were conducted by telephone or via Zoom and were about an hour in duration.

### Participants

Thirty-one participants (14 females and 17 males; mean age = 50 years) involved in treatment (alcohol = 23 participants; drug use = 6 participants, not specified = 1 participant) volunteered to take part in the study by engaging in survey collection after participation in SMART Recovery groups. All group participants were invited to participate in a qualitative interview and 10 elected to do an interview with a member of the research team. Sample size was determined by group size, attendance, and willingness to participate in research. There was no prior established relationship between participants and researchers.

### Measures

Demographic data was collected including: age, gender (tick box: “Male”, “Female”, “other”), and primary drug of concern (“What drug/alcohol are you most concerned about?”). Demand was measured by calculating the average number of participants per group.

The quantitative survey developed for this pilot study was adapted from the 4-item Treatment Effectiveness Assessment [19]. The survey assessed how much participants felt they had improved since their last SMART Recovery group in terms of five key domains: (1) AOD use (“*How much better or worse is your drug/alcohol use?*”); (2) Physical health (“*How much better or worse is your physical health?*”); (3) Mental health and wellbeing (“*How much better or worse is your mental health and wellbeing?*”); (4) Personal responsibilities (“*How much better or worse are you in taking care of your responsibilities*?”); and (5) Feelings of connection with others (“*Do you feel you are more connected with others?*”). Participants assessed these statements on an adapted 11-point scale, from -5 (much worse) to + 5 (much better) with 0 representing no change. Three additional questions were also asked to assess participants’ perceived benefits of attending SMART Recovery: (1) “*I have benefited from attending SMART Recovery in terms of managing problem behaviours/issues*”; (2) “*I feel more able to cope with life's challenges since attending SMART*”; and (3) “*I felt supported by other members in today’s group*”. A final question about participants’ confidence in creating a plan was not included in the online version of the questionnaire and thus is not included in this report. Responses were on an 11-point scale from ‘0’ = ‘completely disagree’ through to ‘10’ = ‘completely agree’. Quantitative results were calculated based on data collected from the final attendance for each participant.

Ten participants also completed semi-structured qualitative interviews, which were guided by an interview schedule. The interview schedule was developed based on our reading of the literature and through discussion amongst authors. The schedule included 8 open-ended questions, along with prompts, exploring participants’ experiences of the SMART Recovery pilot (e.g., “Could you tell me a little bit about your experience of participating in SMART?”, “Can you describe your experiences with the other attendees?”, “What were your thoughts about the group being connected with a treatment service?”, etc.).

### Data analysis

Survey data was analysed using descriptive statistics (frequency, means) and single-sample t-tests, using SPSS version 20 IBM. Audio-recordings of interviews were transcribed verbatim and uploaded into the NVivo qualitative data management program for analysis. Thematic analysis [9], was used to analyse qualitative interview data. This involved familiarisation with the data, coding data, developing and refining themes and interpretation through discussion amongst the team, and selecting illustrative quotes using pseudonyms to preserve confidentiality.

## Results

### Quantitative

A total of 75 SMART Recovery groups were run by the pilot sites, with a total of 486 attendances (138 face-to-face and 348 online, an average attendance of 6.5 people per meeting). Not all attendances were unique with people often attending groups regularly. Attendees from the face-to-face groups were invited to take part in the study, with 31 participants agreeing.

**Fig. 1:**
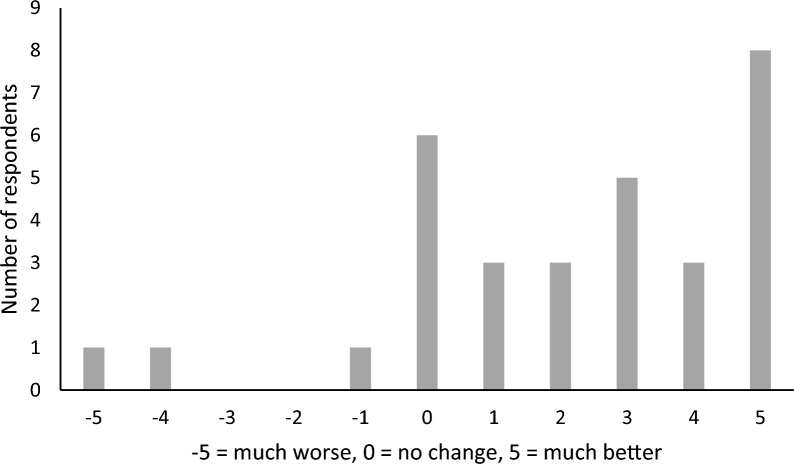
Reported changes in AOD use since attending SMART Recovery groups. Of the 6 participants who reported “no change”, 4 reported they were abstinent from their primary drug of concern and only 2 reported consumption of their PDOC in the preceding 7 days

In total, 71% of participants reported a reduction in alcohol/drug use since their previous SMART Recovery meeting, with 19% reporting no change (see Fig. 1). Importantly the majority of those who reported no change were abstinent, with only 2 participants reporting no change who were not abstinent from their primary drug of concern (PDOC). Thus, 94% of respondents reported a reduction in use, or maintenance of abstinence, of their PDOC. A single sample t-test against a hypothetical mean of zero, which would indicate no change, revealed that participants’ consumption of their PDOC significantly reduced (*t*(30) = 4.49, *p* < 0.001).

**Fig. 2:**
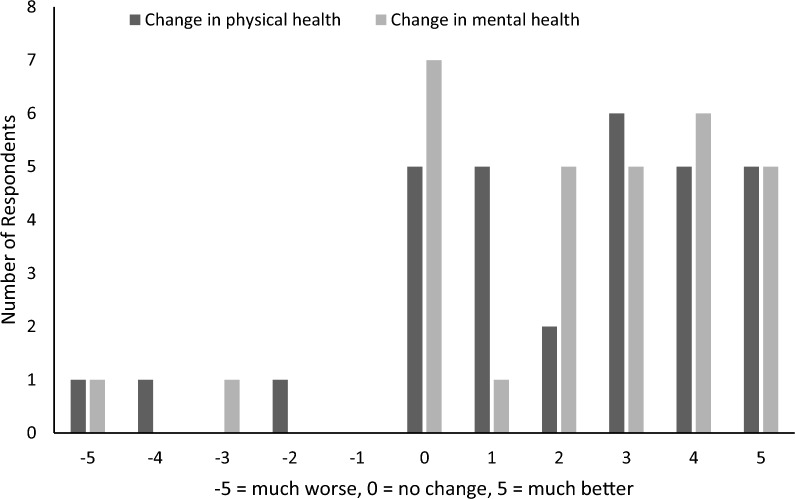
Reported changes in physical and mental health and wellbeing. Change in physical health reflects answers to the question “Since your last SMART group, how much better or worse is your physical health?”. Change in mental health reflects answers to the question “Since your last SMART group, how much better or worse is your mental health and wellbeing?”

In other domains, 74% reported a positive change in their physical health and 71% reported a positive change in their mental health and wellbeing since their last SMART Recovery meeting (see Fig. 2). Single sample t-tests against a hypothetical mean of zero, which would indicate no change, revealed that participants’ physical health (*t*(30) = 4.28, *p* < 0.001) and mental health and wellbeing (*t*(30) = 4.97, *p* < 0.001) significantly improved.

**Fig. 3:**
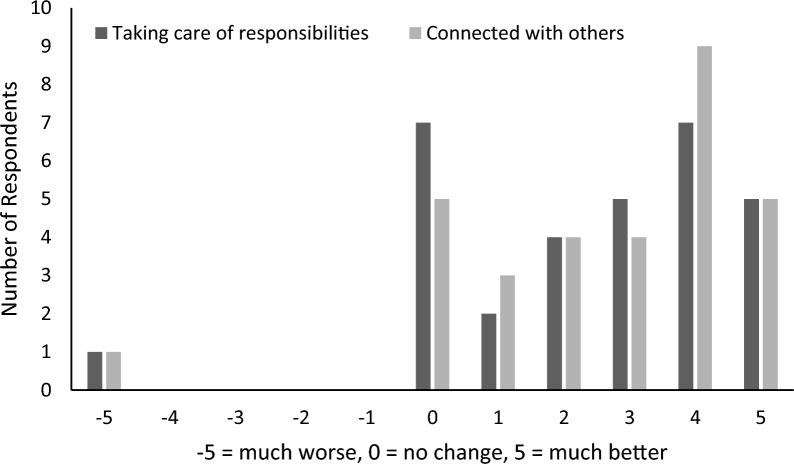
Reported changes in personal responsibilites and connectedness. Taking care of responsibilities reflects answers to the question “Since your last SMART group, how much better or worse are you in taking care of your personal responsibilities?”. Connected with others reflects answers to the question “Since your last SMART group, do you feel more connected with others?”

Similar trends were noted in respect to lifestyle factors with 74% reporting that they were better able to take care of their personal responsibilities, and a single sample t-test (against a value of zero) revealed this improvement was significant (*t*(30) = 5.80, *p* < 0.001). Further, 81% reported they were better connected with others since their last SMART Recovery meeting (see Fig. 3), with a single sample t-test (against zero) revealing this improvement was also significant (*t*(30) = 6.40, *p* < 0.001). Encouragingly, only 1 participant reported a decline in personal responsibilities and feeling more disconnected from others.

**Fig. 4:**
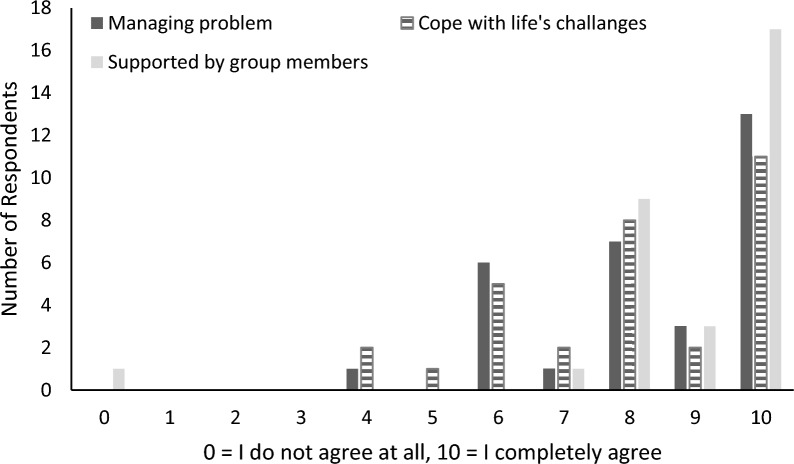
Participants level of agreement with post meeting statements

Responses to the three additional items showed participants significantly agreed with all three statements, with 97% indicating that they could better manage problematic substance use (*t*(30) = 10.81, *p* < 0.001), 90% that they were better able to cope with life’s challenges (*t*(30) = 8.86, *p* < 0.001), and 97% that they felt supported by members of the group during the meetings (*t*(30) = 11.31, *p* < 0.001; Fig. 4). Single sample t-tests for these three measures, which had response options ranging from 0 to 10, were tested against a hypothetical mean of 5, which would indicate a neutral position (i.e. neither agreeing nor disagreeing with the statement).

### Qualitative responses

**Fig. 5:**
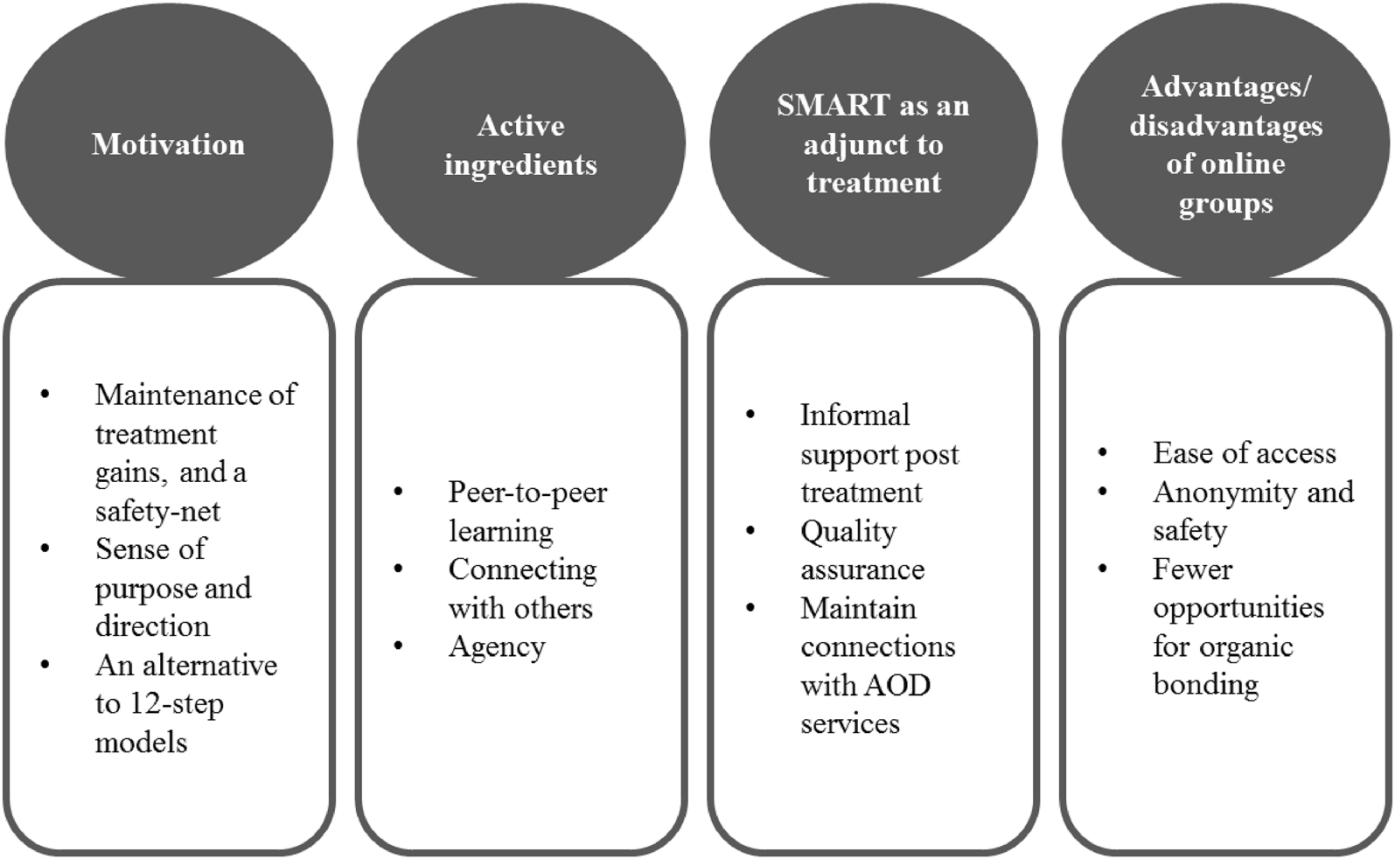
Themes from qualitative interviews

Analysis of qualitative interviews revealed four common themes related to participants’ experiences of SMART, which corroborate and provide further insights to the quantitative component. Further, the qualitative data reveals participants’ experiences of attending SMART run by existing AOD services both online and in-person. The first theme was, *motivation—*participants discussed their reasons for attending SMART Recovery. Secondly, participants highlighted the many *active ingredients* that likely underpin the benefits shown in the quantitative data. Participants also discussed the *integration of SMART into existing AOD services*. Findings from this theme helped to explain why uptake was strong and how the integration of SMART into AOD services conferred multiple benefits. Finally, due to the transition to online halfway through the study, many participants commented on the *advantages and disadvantages of online groups*. As illustrated in Fig. 5, several sub-themes were identified.

### Motivation

As illustrated in Fig. 5, participants were *motivated* to attend SMART Recovery (theme 1) for a number of different reasons. Some felt that formal treatment was no longer relevant to them (e.g., their AOD use had stabilised), and they viewed SMART Recovery as an informal support that they could use, as a ‘safety-net’ if needed.

*“For me I use it mainly just for lifestyle and wellbeing right now, but I know if I were to have cravings or anything or thoughts like that, I know I'd be able to talk about that.” (Gregory)*

*“I just needed to have something to fall back on if I started to struggle.” (Kylie)*

Others felt that the program provided them with the opportunity to focus on the ‘bigger picture’ beyond substance use (e.g., learning to function in everyday life). SMART provided participants with an opportunity to actively engage in their recovery, supported self-efficacy and was a welcome alternative to 12-step programs, due to its secular nature, flexible approach and focus on broader life goals.

*“The reason I got into this group was for recovery and I did it to live life, and it’s not just about the behaviour of using, I have to learn to live life again and that entails everything. So a group like SMART, I’ve really been able to talk a bit more and focus a bit more about life and how to do that now I’m sober, because it’s really different, even interacting with people and talking with people, having a conversation, going to the shops and paying bills, walking out the front to go to the letterbox that was something I couldn’t do.” (Linsey)*

*“I don’t really get into the higher power thing, and I think NA has a tendency to get off topic and people just sharing war stories, and it's more about using, and sometimes it can trigger me I guess. I prefer SMART how it’s all goal based and stuff.” (Gregory)*

Another participant recognised that attending SMART Recovery gave a sense of purpose, perhaps through helping others in their recovery journeys or by providing structure to their week.

*“I really have benefited. It gives me purpose. While I’m unemployed, when you have a substance abuse problem you have no purpose in life. SMART gives me purpose. But also you have to restart.” (Esme)*

Participants also indicated that they liked the harm-reduction focus of SMART Recovery and its embracing of behaviour change at all levels, recognising that recovery is not a linear journey and that an individuals’ goals may change over time.

*“I believe that I’m probably alive because of SMART. I believe stopping drinking and reducing drinking has preserved me in a way that wouldn’t have happened otherwise, and that’s the important thing about SMART’s harm minimisation focus.” (Joshua)*

*“If you don’t want to be abstinent, if in the future you eventually want to have a glass of wine a week, I’m not in that place at the moment, but you know there is none of that discussion in AA.” (Anton)*

These motivations may have been an important ingredient in the emergence of the benefits demonstrated in the quantitative data, such as participants reporting they felt better able to manage substance use, to cope with life’s challenges, and the non-substance related benefits such as improved wellbeing and physical health. The motivation (theme 1) to attend was in pursuit of the reported benefits. Whereas theme 2 identified the active ingredients underpinning the reported benefits.

### Active ingredients

Participants discussed experiences that likely contributed to the benefits seen in the quantitative data. Participants discussed the opportunities for and benefits of *peer to peer learning* and *social connections* within SMART. Most participants expressed that they gained something from peers such as learning about recovery. The statements tended to place a high sense of worth on what was, or could be learnt from peers, due to their rich life experiences.

*“What I like about SMART is there’s that diversity, so there’s different life experiences that people talk about that you can learn from.” (Ben)*

*“Every week I get something out of it, what other people suggest, even if it’s just a phrase or an idea, I take that away." (Esme)*

Participants seemed to value connection with other group members highly, due to a feeling that the members of the group were being genuine, and as a way to connect and overcome loneliness. It was apparent that many participants attended the group to establish connections with others, for some in which they were unable to do with their personal networks because they felt they could be more honest with the members of SMART or because they had to distance themselves from friends who were actively using substances. At a base level, SMART groups circumvented feelings of isolation.

*“I would recommend it to anyone who even just feels lonely. Addiction can be a very isolating thing.” (Linsey)*

*“Everyone has been really open and willing to share about everything, no one was hiding behind anything, you could feel it was genuine and there was trust. …it’s easier to talk to people who aren’t your close friends.” (Ben)*

*“I really appreciate the ability to just connect with people.” (Linsey)*

*“You have to restart your life all over again because obviously you have to wipe everyone that’s a user, or it's handy to. So it’s the positive connections you get out of the group." (Esme)*

As illustrated above many indicated that groups foster social connection and trust.

Participants developed the capacity to manage and drive their own recovery, providing a sense of *agency*.

*“I like the fact that if I’m abstaining, I get support, but if I change my mind and I want to try and manage my drinking, I get support—I’m not left alone.” (Joshua) who also said “I’ve been challenged about trying to modify my drinking and that’s probably been helpful.” (Joshua)*

Further illustrating the subtheme of agency was the common perception that individual goal setting was helpful, and that its iterative nature and focus on small, achievable objectives helped to build confidence and motivation. Feedback from peers and facilitators could help adjust goals that were not met to make them more achievable, further fostering agency.

*“I also think just having that short-term goal and planning for the week ahead and then coming back and talking about how you went and what you can do differently. Like it's really easy to make small adjustments and move forward.” (Gregory)*

The active ingredients of SMART Recovery groups that emerged from the qualitative data such as peer-to-peer learning, connection with others and agency (particularly around goal setting) likely underpin many of the reported benefits from the quantitative data, e.g., feeling better able to cope with life’s challenges, taking care of personal responsibilities, feeling better connected with others, and improved mental health as well as reduced substance use.

### SMART as an adjunct to formal treatment

This theme covers participants’ views on the integration of SMART into formal AOD treatment and addresses research question (iii). Several participants noted how SMART Recovery represents an informal source of support that intersects with and complements formal treatment.

*“Those medical services and individual counsellors you’re coming across, the stuff you’re learning in SMART is not too different to that, there might be more acceptance and commitment therapy in some of those services than there is in SMART, but it slots in very nicely with those and the medical model.” (Joshua)*

Having the SMART Recovery group embedded within the AOD treatment system provided a level quality assurance.

*“With non-SMART groups there aren’t the same checks and balances on things compared to if you were employed at a professional service.” (Joshua)*

*“I’ve been a client of [service name removed] for years and also given consumer feedback so…I know the kind of high-quality organisation it is, which adds a great deal of weight to their meeting. It’s like a guarantee almost, of quality.” (Elizabeth)*

This also provided the opportunity to maintain the relationships they had developed during the earlier stages of their recovery. Knowing that their trusted clinicians/peer workers were running groups, motivated some participants (particularly those new to SMART Recovery, or who experienced high anxiety) to attend and contribute to group discussion.

*“I think because you’re a part of the service you kind of have a different relationship with the facilitators...but [name removed] isn’t just a facilitator she’s also a worker for me and I’ve known [name removed] since 2016, so I have a relationship with them...With SMART the relationships are there. There are benefits to that – they know when I don’t want to talk and when to push.” (Julia)*

Several participants also acknowledged that they would not have attended SMART Recovery were it not for the pilot, even if they had prior knowledge of its existence in the community.

*“I probably wouldn’t have been aware of it if it was a free-standing group. I think for that reason and others it's good that it was connected to [service name removed]. Yeah you don’t know what’s out there until someone says “oh you should go to this group” you know?" (Esme)*

### Advantages and disadvantages of online groups

Finally, a common theme that emerged was around participants perceived advantages and disadvantages of online groups. Many recognised the affordances of online groups, including the convenience in terms of ease of access (e.g., not needing to travel to a venue), which enabled people in regional areas to attend.

*“I wouldn’t be able to go to the group and get the support I need if they weren’t online so it would be a shame if they didn’t carry on.” (Joshua)*

*“There is no current SMART meeting in Geelong. I wouldn’t be able to do [service name]’s meeting face to face because you’re in Melbourne and I’m in Geelong! It’s a two train trip!” (Elizabeth)*

Others appreciated the greater anonymity and safety, particularly for those who might be new to peer support, or anxious about attending an in-person group:

*“I think zoom is just as effective. I think that I’m an extravert and I like to be around people and I like face-to-face [meetings], but I had severe anxiety when I first got clean, so Zoom was perfect for me because it wasn’t so daunting…. but for where I am now, I prefer face-to-face, but it could be very daunting for people who are early in recovery and really anxious. Zoom may be good for them." (Esme)*

However, several also noted the advantages of face-to-face meetings included opportunities for more organic bonding and connections with group members that are not easily emulated online, where discussion is more stilted.

*“We got chatting a bit beforehand in face-to-face groups so there’s that social interaction that’s not part of the actual group whereas with the remote group we just wait silently on mute until everyone is in the room and there isn’t that social element to it before or after we kick off.” (Ben)*

*"I think having the option of both online and face-to-face groups would be good, but for me who wants a social connection maybe there’s a bit more of that in the face-to-face groups." (Ben)*

## Discussion

The study aimed to pilot the integration of SMART Recovery into AOD services to determine its uptake, impact, and to explore client experiences. Answering research question (i), group attendance rates of 6.5 per meeting indicate that there was reasonable demand of SMART when integrated into existing AOD services. This uptake is similar to the average of 6 attendees per meeting reported on the SMART Recovery Australia website (SMART Recovery Australia, accessed 2022). Importantly, the qualitative results suggest that the integration of SMART into existing services may introduce SMART Recovery to those reluctant to attend community run groups. Addressing the question of participant benefits (research question ii), more than nine out of ten participants reported positive substance use outcomes (either maintenance of abstinence or reduction in substance use). At least 70% reported improved physical health, ability to take care of their personal responsibilities, connection with others, and mental health and wellbeing since their last SMART Recovery meeting. These findings support previous findings that peer support groups bolster treatment effects and enhance long-term recovery from AOD use [15, 17, 23]. Further, the current findings confirm previous research trials and quasi-experimental studies demonstrating the benefits of attending SMART Recovery [1, 7, 18, 26, 35], and extend these findings to SMART groups integrated into existing AOD treatment. The qualitative data is consistent with these findings and reveal potential mechanisms behind the reported benefits; showing that participants found groups beneficial in providing ongoing support, connection with others, and autonomy of their own recovery.

Importantly, the qualitative data suggested the addition of SMART Recovery provided pro-recovery activities and connections that transcended what could be provided by traditional treatment. Participants discussed the value in hearing and learning from the life experience of others, of being open with peers, of goal setting within the group, and of social connection. These findings support the assertion that peer support facilitates hope, social connection and legitimises the efficacy of new coping skills [16]. The findings are also consistent with two (‘improving coping skills’ and ‘bonding and support’) of the five components of addiction recovery groups identified by Rettie et al. [27]. Participants also highlighted the benefits of SMART being run by established services, which provided a level of quality assurance and continuity of care. The qualitative results indicated that whilst online groups had its advantages in terms of increased access, anonymity and convenience, the deeper connection that comes from in-person meetings, meant face-to-face groups were favoured by others. These findings largely reflect previous research showing that online groups foster personal empowerment, but the social distance can be a disadvantage for many [3, 5, 34].

It is important to acknowledge some of the limitations of the study, largely the change in data collection methods which was necessitated by the need to run online meetings to accommodate COVID-19 related restrictions. Importantly the absence of a control group (i.e., people not attending SMART Recovery), means the aforementioned improvements cannot be directly attributed to SMART Recovery attendance itself and cannot be disentangled from generic treatment effects and other factors impacting on substance use, health and wellbeing. Further, baseline, pre-group data was not collected so judgments of improvements are retrospective and thus, subject to memory biases and social desirability effects. Additionally, those who agreed to participate in the study and who returned the survey likely represented the participants most engaged in SMART recovery, which could positively bias the findings. Finally, we must exercise caution generalising results to the broader treatment seeking population since then proportion of clients who attended meetings and participated in the survey was not known. Nonetheless, the data illustrate the uptake of SMART Recovery within existing AOD services and the multiple ways in which AOD clients benefited. Further, the qualitative data supported these findings and provided potential active ingredients behind these benefits. Future research should investigate the effectiveness of the integration of SMART Recovery in AOD treatment relative to a control group or other form of peer support in a statistically-powered, randomised control trial.

The results of the current paper highlight the benefits of SMART Recovery and echo the findings of the limited research on the effectiveness of SMART Recovery [7, 12–14, 16, 18, 35]. In light of these demonstrated benefits for clients and the fact that groups were run by 1–2 clinicians/peers with up to 18 clients per meeting, it is likely that SMART Recovery offers a cost-effective model for supporting multiple clients. Future research, especially RCTs should include methods to determine its cost-effectiveness, as has been established with AA [17]. In conclusion, in light of our consistent quantitative and qualitative findings, it appears people in treatment may derive additional benefit from attending SMART Recovery, not only better management of their AOD use, but in their health, wellbeing and sense of connection with others.

### Preconception

One of the authors was familiar with SMART Recovery, having experience as a SMART Recovery facilitator, and had positive preconceptions about SMART Recovery. While familiarity with a field can be a resource, preconceptions can influence assumptions and interpretations in a way that researchers are unaware of [6]. To mitigate potential bias, qualitative and quantitative analyses were conducted by two other researchers who have had no previous experience with SMART Recovery facilitation or research. The authors believe this step has mitigated any potential bias.

## Data Availability

The qualitative datasets generated and/or analysed during the current study are not available due to the sensitive nature of their content and ethical limitations on data sharing.

## Abbreviations

AOD: Alcohol, and/or other drug-related
AA: Alcoholics anonymous
NA: Narcotics anonymous
CBT: Cognitive behaviour therapy
